# Relationship among coronary plaque compliance, coronary risk factors and tissue characteristics evaluated by integrated backscatter intravascular ultrasound

**DOI:** 10.1186/1476-7120-10-32

**Published:** 2012-07-30

**Authors:** Yoshiyuki Ishihara, Masanori Kawasaki, Arihiro Hattori, Hajime Imai, Shigekiyo Takahashi, Hironobu Sato, Tomoki Kubota, Munenori Okubo, Shinsuke Ojio, Kazuhiko Nishigaki, Genzou Takemura, Hisayoshi Fujiwara, Shinya Minatoguchi

**Affiliations:** 1 Department of Cardiology, Gifu University Graduate School of Medicine, 1-1 Yanagido, Gifu, 501-1194, Japan

**Keywords:** Coronary artery disease, Intravascular ultrasound, Plaque, Stiffness, Tissue

## Abstract

**Background:**

The purpose of the present study was to evaluate the mechanical properties of coronary plaques and plaque behavior, and to elucidate the relationship among tissue characteristics of coronary plaques, mechanical properties and coronary risk factors using integrated backscatter intravascular ultrasound (IB-IVUS).

**Methods:**

Non-targeted plaques with moderate stenosis (plaque burden at the minimal lumen site: 50-70%) located proximal to the site of the percutaneous coronary intervention target lesions were evaluated by IB-IVUS. Thirty-six plaques (less calcified group: an arc of calcification ≤10°) in 36 patients and 22 plaques (moderately calcified group: 10° < an arc of calcification ≤60°) in 22 patients were evaluated. External elastic membrane volume (EEMV) compliance, lumen volume (LV) compliance, plaque volume (PV) response (difference between PV in systole and diastole), EEM area stiffness index were measured at the minimal lumen site. Relative lipid volume (lipid volume/internal elastic membrane volume) was calculated by IB-IVUS.

**Results:**

In the less calcified group, there was a significant correlation between EEMV compliance and the relative lipid volume (r = 0.456, p = 0.005). There was a significant inverse correlation between EEM area stiffness index and the relative lipid volume (p = 0.032, r = −0.358). The LV compliance and EEM area stiffness index were significantly different in the diabetes mellitus (DM) group than in the non-DM group (1.32 ± 1.49 vs. 2.47 ± 1.79%/10 mmHg, p =0.014 and 28.3 ± 26.0 vs. 15.7 ± 17.2, p =0.020). The EEMV compliance and EEM area stiffness index were significantly different in the hypertension (HTN) group than in the non-HTN group (0.77 ± 0.68 vs. 1.57 ± 0.95%/10 mmHg, p =0.012 and 26.5 ± 24.3 vs. 13.0 ± 16.7, p =0.020). These relationships were not seen in the moderately calcified group.

**Conclusion:**

The present study provided new findings that there was a significant correlation between mechanical properties and tissue characteristics of coronary arteries. In addition, our results suggested that the EEMV compliance and the LV compliance were independent and the compliance was significantly impaired in the patients with DM and/or HTN. Assessment of coronary mechanical properties during PCI may provide us with useful information regarding the risk stratification of patients with coronary heart disease.

## Introduction

Atherosclerotic changes consist of both a structural (atherosis) and functional (sclerosis) component. A pathological study in elastic arteries such as the carotid artery has shown that sclerotic changes are reflected by a decrease in vessel extensibility due to the degeneration of elastic and collagen fibers, whereas atherotic changes are reflected by an increase in plaque volume [1]. However, relationship between tissue components and the mechanical properties of coronary arteries with atheromatous changes have not been adequately investigated.

Intravascular ultrasound (IVUS) allows cross-sectional imaging of coronary arteries and provides a comprehensive assessment of atherosclerotic plaques in vivo [2,3]. We previously reported that integrated backscatter (IB)-IVUS had high sensitivity and specificity (90-95%) for the tissue characterization of coronary plaques using histology as a gold standard [3-6]. In those studies, we constructed two-dimensional (2D) or three-dimensional (3D) color-coded maps of plaque components based on the IB values [4-9].

The purpose of the present study was to evaluate the mechanical properties of coronary arteries and plaque behavior, and to elucidate the relationship among mechanical properties, tissue characteristics of coronary plaques and coronary risk factors using IB-IVUS.

## Methods

### Subjected patients and coronary plaques

We enrolled 150 consecutive patients with stable angina pectoris that underwent percutaneous coronary intervention (PCI) to the left anterior descending arteries and left circumflex arteries. Right coronary arteries were excluded because they often have ectasia that results from medial replacement of smooth muscle cells with hyalinized collagen and this is not typical coronary atherosclerotic lesions [10]. Patients were excluded if they had unstable angina or myocardial infarction within the previous 3 months, an ejection fraction ≤30%, atrial fibrillation or frequent ventricular premature beat. Patients with low systolic pressure (≤100 mmHg) during catheterization were also excluded. Non-targeted plaques with moderate stenosis (plaque burden at the minimal lumen site: 50-70%) located proximal to the site of the PCI target lesions were evaluated, because previous IVUS studies demonstrated that there is a significant relationship between plaque burden and arterial luminal compliance [11,12].

**Figure 1:**
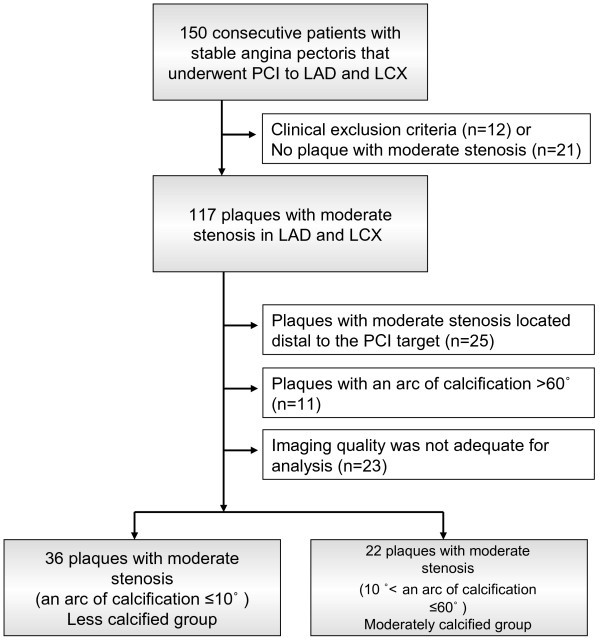
** Study flow chart.** LAD: left anterior descending artery. LCX: left circumflex artery. PCI: percutaneous coronary intervention.

Proximal lesions in the left anterior descending arteries and left circumflex arteries (#6 and #11) were selected, since the previous study demonstrated that proximal and distal pressures in atheromatous coronary arteries without clinically significant stenosis vary in pressure by 5 mmHg [13]. Plaques with an arc of calcification >60° were excluded because acoustic shadow due to calcification affected rigorous measurement of plaque and vessel area. Plaques which imaging quality was not adequate for analysis were also excluded. Finally, thirty-six plaques (less calcified group: an arc of calcification ≤10°) with moderate stenosis (plaque burden at minimal lumen site: 50-70%) in 36 patients and 22 plaques (moderately calcified group: 10° < an arc of calcification ≤60°) with moderate stenosis (plaque burden at minimal lumen site: 50-70%) in 22 patients were analyzed (Figure 1). Risk factors for coronary artery disease were evaluated in enrolled patients, including hypertension (HTN) (medication-dependent or systolic BP ≥140 and/or diastolic BP ≥90 mmHg), type 2 diabetes mellitus (DM) (medication-dependent or hemoglobin (Hb) A1c ≥ 6.5%), dyslipidemia (DL) (medication-dependent, LDL cholesterol ≥140 mg/dl and/or HDL cholesterol <40 mg/dl) and current smoking. The protocol was approved by the institutional ethics committees, and informed consent was obtained from each patient. 

### Integrated backscatter intravascular ultrasound system and data acquisition

An IVUS imaging system (VISIWAVE, Terumo, Japan) was used to obtain cross-sectional IB-IVUS images. Ultrasound backscattered signals were acquired using a 38 MHz mechanically-rotating IVUS catheter (ViewIT, Terumo, Tokyo, Japan). The details of the system and its clinical usefulness have been reported previously [6-9,14]. Electrocardiographic (ECG) data were continuously displayed on conventional IVUS images. The values for systemic pressure were obtained from the tip of the guiding catheter (6–7 Fr) in the ostium of the left main coronary trunk and were continuously displayed on the monitor. All vasoactive medications were discontinued at least 12 hours before catheterization.

We administered an optimal dose of intracoronary isosorbide dinitrate before the measurements for the prevention of coronary spasm. The IVUS catheter was advanced into the coronary artery and IB-IVUS images were acquired at the site of plaques which plaque burden at the minimal lumen site was within 50-70%. All measurements were performed 3–5 minutes after the administration of isosorbide dinitrate, since vasoactive medication can affect coronary distensibility for at least two minutes [15,16].

**Figure 2:**
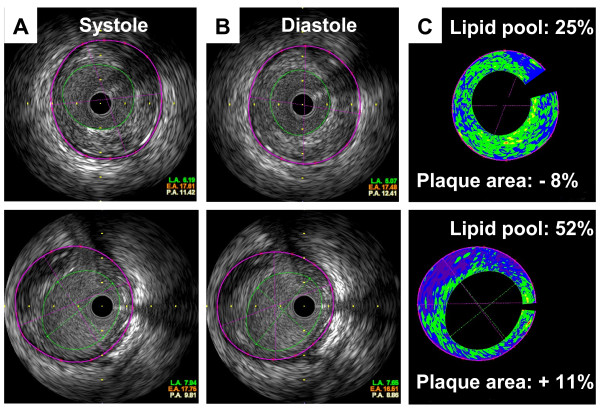
** Intravascular ultrasound images of coronary arteries. (A)** Gray scale images in systole. **(B)** Gray scale images in diastole at the same site as (**A**). **(C)** Integrated backscatter intravascular ultrasound images at the same site as (**A**) and (**B**). **(Upper)** Representative cross-sectional images that showed reduction of plaque area from diastole to systole. **(Lower)** Representative cross-sectional images that showed increment of plaque area from diastole to systole.

The position and axis of IVUS might have slightly changed during the cardiac cycle, preventing rigorous measurement of coronary compliance. Therefore, we performed volumetric analysis using three cross-sections (0.5 mm proximally, and distally to the site of minimal lumen diameter). That is, thickness of analyzed lesion was one millimeter. Cross-sectional images were quantified for lumen volume (LV), external elastic membrane volume (EEMV), and plaque volume (PV = EEMV - LV) by use of software included with the IVUS system (Figure 2). Total EEMV, PV and LV was calculated as the sum of three cross-sections (0.5 mm proximally, and distally to the site of minimal lumen diameter). The eccentricity rate was calculated as (maximum plaque plus media thickness - minimum plaque plus media thickness)/maximum plaque plus media thickness. The remodeling index was defined as the ratio of EEM area at the measured lesion to average of EEM area at the proximal reference site and EEM area at the distal reference site. We determined the maximum and minimum EEMV and LV between the onset of the QRS complex (end-diastole) and the termination of the T-wave (end-systole), since these criteria were used in previous studies [10,11,17]. 

Four mechanical properties including pressure-independent vascular stiffness index were calculated using the following formulas:

(1) Corrected EEMV compliance (%/10 mmHg) = [(EEMV at systole – EEMV at diastole)/EEMV at diastole]/(SBP-DBP) x 10, where SBP is the blood pressure in systole and DBP is the blood pressure in diastole

(2) Corrected LV compliance (%/10 mmHg) = [(LV at systole – LV at diastole)/LV at diastole]/(SBP-DBP) x 10

(3) Corrected PV response (%/10 mmHg) = [(PV at systole – PV at diastole)/PV at diastole]/(SBP-DBP) x 10

(4) EEM area stiffness index = [In (SBP/DBP)]/[(maximum EEM area - minimum EEM area)/minimum EEM area]

The EEM area stiffness index was calculated as the average of the EEM area stiffness index at three cross-sections (every 0.5 mm proximally, and distally to the site of minimal lumen diameter). Determination of the stiffness index requires measurement of vessel diameter [18,19]. In the previous studies, the diameter of coronary arteries is calculated by vessel area assuming the cross-section is circular. However, we measured the stiffness index using vessel area since we could not be certain of a circular geometry.

### Construction of 3D IB-IVUS images

The 3D construction by connecting consecutive 2D IB-IVUS images was automatically performed by computer software (T3D, Fortner Research LLC). We also constructed a 3D plaque response map by changing color range of the 3D IB-IVUS color-coded maps. Of the 36 lesions evaluated, 3D color-coded maps were constructed for five plaques which plaque burden of 17 cross-sections (every 1 mm axial interval for 8 mm proximally, and distally to the site of minimal lumen diameter) were within 50-70% by connecting the results of 17 cross-sections.

### Reproducibility and reliability of HU measurements

We determined the interobserver variability of LV using 20 randomly-selected LV and EEMV that were measured by two observers in a blinded way. Likewise, we determined the intraobserver variability of LV and EEMV using 20 randomly-selected cross-sections that were measured twice by one observer with a 7-day interval between the two measurements. We determined the standard deviation of relative lipid area during the cardiac cycle using 20 randomly-selected cross-sections.

### Statistical analyses

Data are reported as mean ± standard deviation (SD). Normality of distribution was tested using the Kolmogorov-Smirnov test. The significance of the differences between groups that were normally distributed and had similar variances was tested by an unpaired Student’s *t* test. Otherwise, Mann–Whitney *U* test was used to compare the difference between groups. Categorical data were summarized as percentages and compared using a Chi-square test or Fisher exact test. The relationships between the mechanical properties and the relative lipid pool were tested for significance by linear regression analysis. A p value <0.05 was considered statistically significant. Statistical analyses were performed using Stat View version 5.0 (SAS Institute Inc, Cray, NC).

## Results

### Patient characteristics

All patients underwent IB-IVUS analysis in non-target lesions without any complications. The patients’ characteristics are shown in Table 1. Age and medication with statin in the moderately calcified group were significantly higher than in the less calcified group. The total cholesterol level and LDL cholesterol level in the less calcified group was significantly higher than those in the moderately calcified group because of the higher rate of medication with statin in the moderately calcified group. At the time of measurement, systolic pressure in the ostium of left main coronary trunk ranged from 103 to 191 mmHg, and diastolic pressure ranged from 48 to 97 mmHg (Table 1).

**Table 1:** Clinical and laboratory characteristics

	**Arc of calc ≤10°**	**10° < Arc of calc ≤60°**	**p value**
	**(n = 36)**	**(n = 22)**	
Men, %	31 (86)	15 (68)	0.18
Age, y	65 ± 10	72 ± 9	0.007
Clinical history, n (%)			
Myocardial infarction	10 (28)	7 (32)	0.74
Previous coronary bypass graft	1 (3)	1 (5)	>0.99
Hypertension	25 (69)	15 (68)	0.92
Dyslipidemia	23 (64)	17 (77)	0.38
Current smoker	6 (17)	3 (14)	>0.99
Diabetes mellitus type 2	19 (53)	10 (45)	0.59
Target plaque location, n (%)			
LAD	22 (61)	16 (72)	0.37
LCX	14 (39)	6 (28)	
Medication, n (%)			
Anti-platelet medicine	36 (100)	22 (100)	>0.99
Statin	14 (39)	15 (68)	0.030
Calcium channel blockers	16 (44)	8 (36)	0.54
Beta-blockers	6 (17)	4 (18)	>0.99
Insulin	4 (11)	5 (23)	0.42
ARBs or ACEIs	20 (56)	13 (59)	0.79
Laboratory parameter			
Total cholesterol (mg/dl)	205 ± 37	173 ± 27	<0.001
Triglycerides (mg/dl)	160 ± 96	145 ± 58	0.45
HDL cholesterol (mg/dl)	50 ± 9	45 ± 11	0.12
LDL cholesterol (mg/dl)	120 ± 38	98 ± 30	0.003
Hemoglobin A1c (%)	6.6 ± 0.9	6.6 ± 0.8	0.58
C-reactive protein (mg/dl)	0.75 ± 0.51	0.15 ± 0.16	0.16
Blood pressure during catheterization			
Systolic pressure (mmHg)	147 ± 25	149 ± 29	0.79
Diastolic pressure (mmHg)	74 ± 13	74 ± 13	0.37

Values are mean ± SD. Calc: calcification, LAD: left anterior descending, LCX: left circumflex, ARBs: angiotensin II receptor blockers, ACEIs: angiotensin-converting enzyme inhibitors.

NGSP: National Glycohemoglobin Standardization Program.

### Reproducibility and reliability of measurements

The interobserver correlation coefficient and mean differences in LV were 0.99 and 1.4 ± 0.4%, respectively. The interobserver correlation coefficient and mean differences in EEMV were 0.97 and 2.5 ± 1.4%, respectively. The intraobserver correlation coefficient and mean differences in LA were 0.99 and 1.0 ± 0.9%, respectively. The intraobserver correlation coefficient and mean differences in EEMV were 0.98 and 2.1 ± 1.0%, respectively. The standard deviation of the relative lipid volume during the cardiac cycle was 2.1 ± 0.5%, and since there was no variation during the cardiac cycle, we ignored the influence of the cardiac cycle on relative lipid volume.

### Conventional parameters and mechanical properties

There was significant correlations between LDL cholesterol and EEMV compliance and EEM area stiffness index (r =0.454, p =0.005 and r = −0.463, p =0.005, respectively). However, there was no significant relationship between EEMV compliance and HDL cholesterol (p =0.42) and between EEM area stiffness index and HDL cholesterol (p =0.59).

There were no significant differences between the less and moderately calcified groups in the conventional IVUS parameters except for eccentric rate, relative calcification area (Table 2). The EEMV compliance and LV compliance were significantly greater in the less calcified group than those in the moderately calcified group. There were no significant differences in the PV response and EEM area stiffness index between the less calcified group and the moderately calcified group.

**Table 2:** Angiographic and intravascular ultrasound characteristics

	**Arc of calc ≤10°**	**10° < Arc of calc ≤60°**	**p value**
	**(n = 36)**	**(n = 22)**	
Angiographic stenosis, %	43.9 ± 6.9	41.0 ± 5.6	0.092
Systolic vessel volume, mm^3^	15.0 ± 3.8	13.0 ± 3.7	0.054
Systolic lumen volume, mm^3^	5.7 ± 1.5	5.1 ± 2.0	0.19
Systolic plaque volume, mm^3^	9.2 ± 3.0	7.9 ± 2.4	0.069
Diastolic vessel volume, mm^3^	14.1 ± 3.6	12.5 ± 3.8	0.11
Diastolic lumen volume, mm^3^	5.1 ± 1.6	4.8 ± 1.9	0.44
Diastolic plaque volume, mm^3^	9.0 ± 2.9	7.7 ± 2.5	0.095
Plaque burden, %	61.3 ± 7.9	61.4 ± 9.4	0.97
Eccentricity rate	0.68 ± 0.14	0.53 ± 0.18	0.003
Remodeling index	0.98 ± 0.12	0.97 ± 0.08	0.51
Relative lipid volume, %	44.2 ± 12.8	46.2 ± 14.5	0.58
Relative fibrous volume, %	55.6 ± 12.8	51.3 ± 14.0	0.23
Relative calcification volume, %	0.22 ± 0.18	2.50 ± 1.79	<0.001
EEMV compliance, %/10 mmHg	1.01 ± 0.85	0.58 ± 0.54	0.037
LV compliance, %/10 mmHg	1.86 ± 1.71	0.76 ± 0.87	0.007
PA response, %/10 mmHg	0.95 ± 2.28	0.51 ± 0.95	0.39
EEM area stiffness index	22.3 ± 22.9	32.6 ± 26.8	0.12

Values are mean ± SD. Calc: calcification, EEMV: external elastic membrane volume, LV: lumen volume, PA: plaque area.

### Tissue characteristics and mechanical properties of coronary plaques

**Figure 3:**
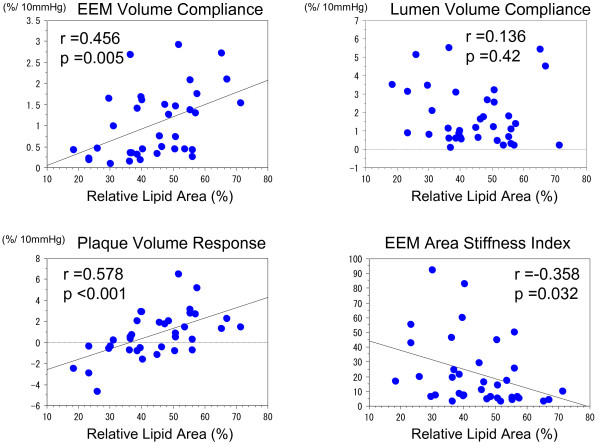
** Correlation among relative lipid volume, mechanical properties and plaque behavior in the less calcified group.** The EEM volume compliance, plaque volume compliance and EEM area stiffness index were significantly correlated with relative lipid area whereas there was no significant correlation between lumen volume compliance and relative lipid area. EEM: external elastic membrane.

**Figure 4:**
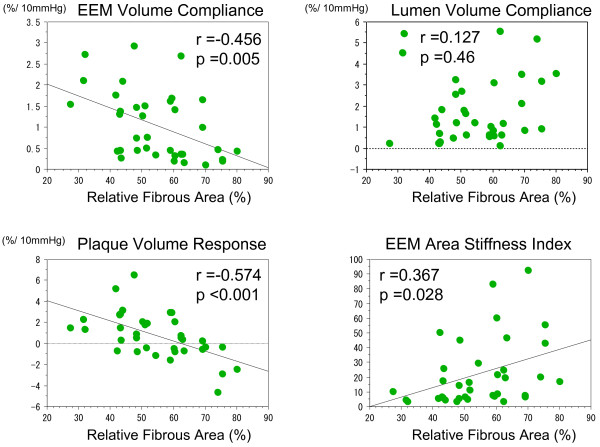
** Correlation among relative fibrous volume, mechanical properties and plaque behavior in the less calcified group.** The EEM volume compliance, plaque volume compliance and EEM area stiffness index were significantly correlated with relative fibrous area whereas there was no significant correlation between lumen volume compliance and relative fibrous area. EEM: external elastic membrane.

**Figure 5:**
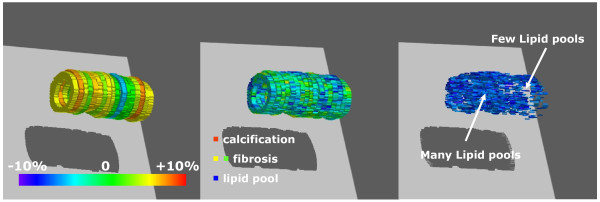
** Three-dimensional intravascular ultrasound images. (Left)** Color-coded map of plaque volume response. Each color corresponded to plaque volume compression and expansion (percentage per 10 mmHg). Light green to orange signified plaque expansion, and dark green to blue indicated plaque compression during cardiac cycle. **(Middle)** Three dimensional integrated backscatter intravascular ultrasound (3D IB-IVUS) color-coded map that showed plaque components. **(Right)** 3D IB-IVUS color-coded map that depicted only lipid pool at the same lesion as the middle image. Note that plaque area response in the lesion with relatively many lipid pools (left side of the segment) was greater than that in the lesion with relatively few lipid pools (right side of the segment).

In the less calcified group, there was significant correlation between LV compliance and EEMV compliance (r =0.390, p =0.019), whereas there was no significant correlation between PV and EEMV compliance (p =0.13). There was a significant correlation between EEMV compliance and the relative lipid volume (r =0.456, p =0.005) (Figure 3). There was a significant inverse correlation between EEMV compliance and the relative fibrous volume (r = −0.456, p =0.005) (Figure 4). The PV was larger in systole when the relative lipid volume was ≥38%, whereas the PV was smaller in systole when the relative lipid volume was <38% (Figure 3). There was a significant correlation between the PV response and the relative lipid volume (p <0.001, r =0.578). There was a significant inverse correlation between the PV response and the relative fibrous volume (p <0.001, r = −0.574). The EEM area stiffness in low relative lipid volume (less than median of the relative lipid volume) was significantly greater than that in high relative lipid volume group (more than and equal to median of the relative lipid volume) (31.1 ± 27.0 vs. 13.7 ± 13.8, p =0.010). There was a significant inverse correlation between EEM area stiffness index and the relative lipid volume (p =0.032, r = −0.358), suggesting that diseased coronary arteries became stiffer when the relative lipid volume was smaller (Figure 5).

In contrast, in the moderately calcified group, there was no relationship between the relative lipid volume and EEMV compliance, PV response, LV compliance and EEM area stiffness index (p =0.13, p =0.21, p =0.92 and p = 0.21, respectively). There was no relationship between the relative fibrous volume and EEMV compliance, PV response, LV compliance and EEM area stiffness index (p =0.15, p =0.25, p =0.90 and p =0.24, respectively). Likewise, there was no relationship between the relative calcification volume and EEMV compliance, PV response, LV compliance and EEM area stiffness index (p =0.31, p =0.30, p =0.88 and p =0.35, respectively).

### Mechanical properties of coronary arteries and coronary risk factors

**Figure 6:**
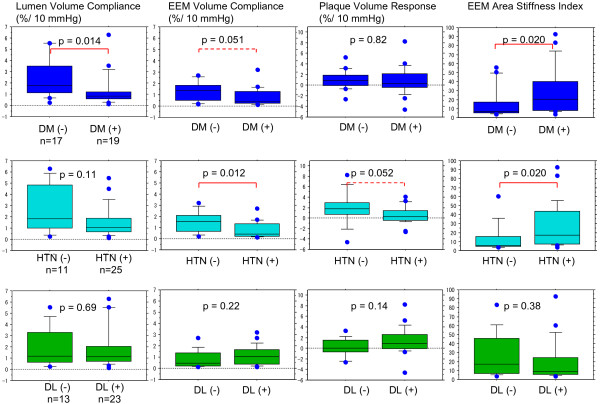
** Relationships between mechanical properties of coronary arteries and coronary risk factors.** DM: diabetes mellitus, HTN: hypertension, DL: dyslipidemia. Non-parametric data was shown in box plot. A line in the box marks: median. Central box spans: 1st quartile and 3rd quartile. Lines extend from the box: 10th and 90th percentile.

We preformed sub-analyses in the less calcified group comparing coronary compliance and stiffness with coronary risk factors (HTN, DM and DL). The results were shown in Figure 6. The LV compliance and EEM area stiffness index were significantly different in the DM group than in the non-DM group (1.32 ± 1.49 vs. 2.47 ± 1.79%/10 mmHg, p =0.014 and 28.3 ± 26.0 vs. 15.7 ± 17.2, p =0.020). The EEMV compliance and EEM area stiffness index were significantly different in the HTN group than in the non-HTN group (0.77 ± 0.68 vs. 1.57 ± 0.95%/10 mmHg, p =0.012 and 26.5 ± 24.3 vs. 13.0 ± 16.7, p =0.020). There was no relationship between the coronary risk factors and tissue characteristics.

## Discussion

We evaluated the mechanical properties and plaque behavior of coronary arteries, and determined that the relationship among tissue characteristics of coronary plaques, mechanical properties and coronary risk factors using IB-IVUS.

### Mechanical properties of coronary arteries and plaque behavior

Several previous studies reported that the compliance of coronary arteries varied among calcified, fibrotic and hypoechogenic plaques that were classified by conventional gray scale IVUS. Nakatani et al. demonstrated that distensibility at angiographically normal coronary sites was significantly correlated with thickness of the intima-media complex [20,21]. Previous intravascular ultrasound studies demonstrated that there was a significant relationship between plaque burden and arterial luminal compliance in diseased coronary arteries with plaques [10,11]. Therefore, in the present study, we limited out analysis to coronary lesions with moderate stenosis (plaque burden: 50-70%) to elucidate the relationship between mechanical properties and tissue components.

In the present study, EEMV and LV were greater in systole than in diastole, and this is consistent with the results of previous studies. However, the increment or reduction of PV (plaque response) depended on the relationship between EEMV compliance and LV compliance. That is, when the increment of EEMV from diastole to systole was greater than that of LV, the PV was greater. In contrast, when the increment of LV from diastole to systole was greater than that of EEMV, the PV was smaller. There was only one previous study showing two possible changes in plaque area (systolic plaque compression or systolic plaque expansion) during the cardiac cycle [17]. In that study, a major determinant of the systolic increase in lumen area in atheromatous coronary lesions was a reduction in plaque area during systole [17]. However, that study did not report that the increment or reduction of PV depended on the relationship between EEMV and LV. The present study demonstrated new insight into the mechanical properties and plaque behavior of coronary arteries.

The present study demonstrated that there was no significant correlation between LV compliance and the relative lipid pool volume, whereas the EEMV compliance was significantly correlated with the relative lipid pool volume. Keymel et al. reported that flow-mediated dilation of brachial arteries as a maker for endothelial function and fractional diameter changes as a maker for mechanical properties of external diameter of the artery independently represented the endothelial function and vascular structure of brachial arteries [22]. A change of LV during cardiac cycle may be affected by the endothelial function that is independent of the change of EEMV and plaque components.

Another intriguing finding in the present study was that the coronary artery was stiffer in the DM or HTN group than in the non-DM or non-HTN group regardless of similar plaque burden and no correlation with tissue characteristics. This finding suggested that coronary sclerotic change due to DM or HTN is diffuse sclerotic process and independent of the change of tissue components that is local atherotic process. A previous study demonstrated that endothelial function and mechanical property of brachial arteries were impaired in patients with DM comparing with patients without DM [22]. Another study showed that aortic stiffness was augmented in phases according to the severity of HTN [23]. However, there have been few studies that demonstrated the relationship between the impairment of mechanical property of coronary arteries and the coronary risk factors. This is the first clinical demonstration of the relationship among mechanical properties, tissue characteristics of coronary plaques and coronary risk factors.

In the moderately calcified group, there was no relationship between the relative lipid volume and EEMV compliance, PV response, LV compliance and EEM area stiffness index. Previous study reported that coronary vessel expansion from diastole to systole in calcified lesions (arc of calcification >25%) was less than that in non-calcified lesions [12]. This is consistent with the results of the present study.

### Clinical implications

The findings in the present study have important clinical implications. It is possible that the plaque response (compression or expansion) demonstrated in the present study could be an alternative index for the dynamic stress on coronary plaques at risk of rupture. Imbalance in these properties within the same coronary plaques might trigger acute coronary syndrome because repetitive plaque deformation during the cardiac cycle increases the dynamic stress on plaques [16,24]. In the present study, LV compliance was significantly impaired and EEM area stiffness increased in the DM group than in the non-DM group. Previous studies demonstrated that coronary endothelial vasodilator dysfunction predicted long-term atherosclerotic disease progression and cardiovascular event rates in the patients with coronary heart disease or hypertension [25,26]. Thus, the assessment of coronary mechanical properties during PCI may provide us with useful information regarding the risk stratification of patients with coronary heart disease.

### Study limitations

There are several limitations of the present study. First, we substituted systemic pressures that were obtained from the tip of the guiding catheter in the ostium of the left main coronary trunk for intracoronary pressures. This substitution may hinder rigorous measurement of coronary compliance. However, we evaluated only lesions in the left anterior descending and left circumflex coronary arteries that were proximal to the PCI target lesions to minimize the pressure difference between the left main coronary trunk and the intracoronary pressures. In addition, the position and axis of IVUS might have slightly changed during the cardiac cycle, preventing rigorous measurement of coronary compliance, although we performed volumetric analysis using three cross-sections. Second, all measurement were performed 3–5 minutes after the administration of isosorbide dinitrate, because the effects of vasoactive medication on coronary distensibility last for at least two minutes [15]. It is possible that other oral medications that patients were on at the time of the study may have influenced coronary compliance. Other factors such as calcification and patient age that might influence coronary compliance should also be considered. Finally, circumferential regional differences in compliance were not considered in the present study because of technical difficulties. Circumferential differences may influence plaque deformation that causes physical stress on plaques resulting in plaque rupture. For the evaluation of regional differences in compliance, elastography is useful [27].

## Conclusions

The present study provided new findings that there was a significant correlation between mechanical properties and tissue characteristics of coronary arteries. In addition, our results suggested that the EEMV compliance and the LV compliance were independent and the compliance was significantly impaired in the patients with DM and/or HTN. Assessment of coronary mechanical properties during PCI may provide us with useful information regarding the risk stratification of patients with coronary heart disease.

## Abbreviations

IB-IVUS: integrated backscatter intravascular ultrasound; LV: lumen volume; EEMV: external elastic membrane volume; PV: plaque volume; HTN: hypertension; DM: diabetes mellitus; DL: dyslipidemia.

## Competing interests

The authors declare that they have no competing interests.

## Authors’ contributions

YI and AH carried out subject recruitment and analyzed data. MK analyzed data and wrote the manuscript. HI, ST, HS, TK, MO and SO performed integrated backscatter ultrasound analysis. KN, GT, HF and SM analyzed data. All authors read and approved the final manuscript.

## References

[B1] Kawasaki M Ito Y Yokoyama H Arai M Takemura G Hara A Ichiki Y Takatsu H Minatoguchi S Fujiwara H Assessment of arterial medial characteristics in human carotid arteries using integrated backscatter ultrasound and its histological implications Atherosclerosis 2005 180 145 154 10.1016/j.atherosclerosis.2004.11.018 15823287

[B2] Nissen SE Yock P Intravascular ultrasound: novel pathophysiological insight and current clinical applications Circulation 2001 103 604 616 10.1161/01.CIR.103.4.604 11157729

[B3] Mintz GS Nissen SE Anderson WD Bailey SR Erbel R Fitzgerald PJ Pinto FJ Rosenfield K Siegel RJ Tuzcu EM Yock PG American College of Cardiology clinical expert consensus document on standards for acquisition, measurement and reporting of intravascular ultrasound studies (ivus). A report of the american college of cardiology task force on clinical expert consensus documents developed in collaboration with the european society of cardiology endorsed by the society of cardiac angiography and interventions J Am Coll Cardiol 2001 37 1478 1492 10.1016/S0735-1097(01)01175-5 11300468

[B4] Kawasaki M Sano K Okubo M Yokoyama H Ito Y Murata I Tsuchiya K Minatoguchi S Zhou X Fujita H Fujiwara H Volumetric quantitative analysis of tissue characteristics of coronary plaques after statin therapy using three dimensional integrated backscatter intravascular ultrasound J Am Coll Cardiol 2005 45 1946 1953 10.1016/j.jacc.2004.09.081 15963391

[B5] Kawasaki M Takatsu H Noda T Sano K Ito Y Hayakawa K Tsuchiya K Arai M Nishigaki K Takemura G Minatoguchi S Fujiwara T Fujiwara H In vivo quantitative tissue characterization of human coronary arterial plaques by use of integrated backscatter intravascular ultrasound and comparison with angioscopic findings Circulation 2002 105 2487 2492 10.1161/01.CIR.0000017200.47342.10 12034654

[B6] Amano T Matsubara T Uetani T Nanki M Marui N Kato M Arai K Yokoi K Ando H Ishii H Izawa H Murohara T Impact of metaboric syndrome on tissue charactreristics of angiographically mild to moderate coronary lesions Integrated backscatter intravascular ultrasound study. J Am Coll Cardiol 2007 49 1149 1156 10.1016/j.jacc.2006.12.028 17367657

[B7] Sano K Kawasaki M Ishihara Y Okubo M Tsuchiya K NishigakiK, Zhou X, Minatoguchi S, Fujita H, Fujiwara H: Assessment of vulnerable plaques causing acute coronary syndrome using integrated backscatter intravascular ultrasound J Am Coll Cardiol 2006 47 734 741 10.1016/j.jacc.2005.09.061 16487837

[B8] Okubo M Kawasaki M Ishihara Y Takeyama U Kubota T Yamaki T Ojio S Nishigaki K Takemura G Saio M Takami T Minatoguchi S Fujiwara H Development of integrated backscatter intravascular ultrasound for tissue characterization of coronary plaques Ultrasound in Med & Biol 2008 34 655 663 10.1016/j.ultrasmedbio.2007.09.015 18077081

[B9] Okubo M Kawasaki M Ishihara Y Takeyama U Yasuda S Kubota T Tanaka S Yamaki T Ojio S Nishigaki K Takemura G Saio M Takami T Fujiwara H Minatoguchi S Tissue characterization of coronary plaques Comparison of integrated backscatter intravascular ultrasound with Virtual Histology intravascular ultrasound. Circ J 2008 72 1631 1639 10.1253/circj.cj-07-0936 18753698

[B10] Virmani R Robinowitz M Atkinson JB Forman MB Silver MD McAllister HA Acquired coronary arterial aneurysms Hum Pathol 1986 17 575 583 10.1016/S0046-8177(86)80129-0 3710470

[B11] Reddy KG Suneja R Nair RN Dhawale P Hodson JM Measurement by intracoronary ultrasound of in vivo arterial compliance within atherosclerotic lesions Am J Cardiol 1993 72 1232 1237 10.1016/0002-9149(93)90289-O 8256697

[B12] Weissman NJ Palacios IF Weyman AE Dynamic expansion of coronary arteries: Implications for intravascular ultrasound measurements Am Heart J 1995 130 46 51 10.1016/0002-8703(95)90234-1 7611122

[B13] De Bruyne B Hersbach F Pijls N Bartunek J Bech JW Heyndrickx GR Gould KL Wijns W Abnormal epicardial coronary resistance in patients with diffuse atheromatous but “normal” coronary angiography Circulation 2002 104 2401 2406 11705815 10.1161/hc4501.099316

[B14] Kawasaki M Hattori A Ishihara Y Okubo M Nishigaki K Takemura G Saio M Takami T Minatoguchi S Tissue characterization of coronary plaques and assessment of thickness of fibrous cap using integrated backscatter intravascular ultrasound Comparison with histology and optical coherence tomography. Circ J 2010 74 2641 8 10.1253/circj.cj-10-0547 20953061

[B15] Akima T Makkar R Nishioka T Dohad S Iida K Goland S Kar S Luo H Siegel RJ Impact of nitroglycerin and verapamil on coronary arterial distensibility as assessed by intravascular ultrasound J Invasive Cardiol 2009 21 162 167 19342754

[B16] Yamagishi M Nissen SE Booth DC Gurley JC Koyama J Kawano S DeMaria AN Coronary reactivity to nitroglycerin: intravascular ultrasound evidence for the importance of plaque distribution J Am Coll Cardiol 1995 25 224 230 10.1016/0735-1097(94)00346-R 7798506

[B17] Shaw JA Kingwell BA Walton AS Cameron JD Pillay P Gatzka CD Dart AM Determinants of coronary artery compliance in subjects with and without angiographic coronary artery disease J Am Coll Cardiol 2002 39 1637 1643 10.1016/S0735-1097(02)01842-9 12020491

[B18] Hayashi K Handa H Nagasawa S Okumura A Moritake K Stiffness and elastic behavior of human intracranial and exracranial arteries J Biomech 1980 13 175 184 10.1016/0021-9290(80)90191-8 7364778

[B19] Hirai T Sasayama S Kawasaki T Yagi S Stiffness of systemic arteries in patients with myocadial infarction A noninvasive method to predict severity of coronary atherosclerosis. Circulation 1989 80 78 86 10.1161/01.cir.80.1.78 2610739

[B20] Alfonso F Macaya C Goicolea J Hernandez R Segovia J Zamorano J Bañuelos C Zarco P Determinants of coronary compliance in patients with coronary artery disease: An Intravascular ultrasound study J Am Coll Cardiol 1994 23 879 884 10.1016/0735-1097(94)90632-7 8106692

[B21] Nakatani S Yamagishi M Tamai J Goto Y Umeno T Kawaguchi A Yutani C Miyatake K Assessment of coronary artery distensibility by intravascular ultrasound Application of simultaneous measurements of luminal area and pressure. Circulation 1995 91 2904 2910 10.1161/01.cir.91.12.2904 7796499

[B22] Keymel S Heinen Y Balzer J Rassaf T Kelm M Lauer T Heiss C Characterization of macro-and microvascular function and structure in patients with type 2 diabetes mellitus Am J Cardiovasc Dis 2011 1 68 75 22254187 PMC3253507

[B23] Tomiyama H Arai T Koji Y Yambe M Motobe K Zaydun G Yamamoto Y Hori S Yamashina A The age-related increase in arterial stiffness is augmented in phases according to the severity of hypertension Hypertens Res 2004 27 465 470 10.1291/hypres.27.465 15302982

[B24] Rothwell PM Villagra R Gibson R Donders RC Warlow CP Evidence of a chronic systemic cause of instability of atherosclerotic plaques Lancet 2000 355 19 24 10.1016/S0140-6736(99)04470-0 10615886

[B25] Schächinger V Britten MB Zeiher AM Prognostic impact of coronary vasodilator dysfunction on adverse long-term outcome of coronary heart disease Circulation 2000 101 1899 1906 10.1161/01.CIR.101.16.1899 10779454

[B26] Perticone F Ceravolo R Pujia A Ventura G Iacopino S Scozzafava A Ferraro A Chello M Mastroroberto P Verdecchia P Schillaci G Prognostic significance of endothelial dysfunction in hypertensive patients Circulation 2001 104 191 196 10.1161/01.CIR.104.2.191 11447085

[B27] Schaar JA de Korte CL Mastik F Strijder C Pasterkamp G Boersma E Serruys PW van der Steen AFW Characterizing vulnerable plaque features with intravascular elastography Circulation 2003 108 2636 2641 10.1161/01.CIR.0000097067.96619.1F 14581406

